# Supplementary material for: Risk of cardiac events with azithromycin—A prediction model
Source: PLoS One. 2020 Oct 15;15(10):e0240379. doi: 10.1371/journal.pone.0240379 (PMC7561086; doi:10.1371/journal.pone.0240379)
Supplement: Attachment — Submitted filename: renamed_226a0.docx [file pone.0240379.s001.docx]

In the paper entitled” Risk of Cardiac Events with Azithromycin – A Prediction Model” by Haridarshan Patel, the authors developed two prediction models, which included baseline demographics, clinical conditions (Model 1), concurrent use of any drug (Model 1) and therapeutic class (Model2) with a risk of QT-prolongation (CQT-Rx) through a splitsample approach, concluded that the Assessment of Cardiac Risk with Azithromycin score maybe helpful to identify patients who are at higher risk of cardiac events following treatment with azithromycin and providers should assess the risk-benefit of using azithromycin and consider alternative antibiotics among high-risk patients.

comments:

1. This is an interesting and fairly well-written paper dealing with identifying patients who are at higher risk of cardiac events following treatment with azithromycin through the Assessment of Cardiac Risk with Azithromycin score.
2. It is well known that ACEIs has protective effect on cardiac remodeling. There is a minor issue that needs attention in order to explain the administration of ACEIs predicts a higher risk of cardiac events following administration with Azithromycin.
