# Supplementary material for: Risk of cardiac events with azithromycin—A prediction model
Source: PLoS One. 2020 Oct 15;15(10):e0240379. doi: 10.1371/journal.pone.0240379 (PMC7561086; doi:10.1371/journal.pone.0240379)
Supplement: Attachment — Submitted filename: Review Comments to the Author responses 7-24-2020.docx [file pone.0240379.s002.docx]

**Review Comments to the Author**

**Editor’s comments**

Please ensure that your manuscript meets PLOS ONE's style requirements, including those for file naming. The PLOS ONE style templates can be found at

<https://journals.plos.org/plosone/s/file?id=wjVg/PLOSOne_formatting_sample_main_body.pdf> and

<https://journals.plos.org/plosone/s/file?id=ba62/PLOSOne_formatting_sample_title_authors_affiliations.pdf>

***Response: Thank you for pointing this out. We have made significant changes based on the journal’s style requirement. Both links were very helpful.***

2. Please include captions for your Supporting Information files at the end of your manuscript, and update any in-text citations to match accordingly. Please see our Supporting Information guidelines for more information: http://journals.plos.org/plosone/s/supporting-information.

***Response: added.***

**Reviewer #1**

Patel et al. in their paper explore the predictors of cardiac adverse event during therapy with azithromycine. Even though azithromycine has been found to be the less pro-arrhythmic macrolide, the arrhythmic risk prediction during therapy with azithromycine has not been extensively investigated previously. Therefore, a tool to predict this risk may have a relevant clinical application. The paper is well written, well conducted, and statistically accurate. The study is conducted on a large population and the number of events are well represented. However, I have some concerns.

***Response: Thank you for the positive feedback. No change required based on this feedback.***

Major concerns

Palpitations is a symptom that may lead to important misleading. It would improve the quality of the paper a better definition of the symptom. For example, a patient whose palpitation is clearly linked to anxiety is not equable to a patient whose palpitation is characterized by sudden onset and termination associated with dizziness. This should be considered a limitation.

***Response: This is helpful and yes, we are in agreement. We believe that palpitation after treatment initiation within 5 days is most likely due to azithromycin use. Although this is a non-specific condition, we are certain that any primary diagnosis related to this condition would be attributable to the treatment, and not just the baseline anxiety. Having said that, we have added this as a limitation of our study (see text in the limitation section):*** *We included palpitation, a non-specific symptom, as one of the cardiac events and assumed that patients experienced such event due to treatment and not because of a baseline condition.*

The mode of death may be important in this context. Indeed, a death due to the underling infectious disease cannot be considered as a cardiac complication of therapy with azithromycine. If the mode of death cannot be explored, this issue should be reported as a limitation.

***Response: We agree that mode of death is important to assess. Our claims database did not have information on the mode of death but rather only occurrence of death in the 5 days after treatment initiation. We have included the following text in our limitation to address this concern:*** *Although we were able to measure the outcome of death using the ICD-9 codes, we lacked information on death occurring in settings other than inpatient hospitalization.*

Minor concerns

Death usually is not a cause of admission. In the abstract I would move “or death” after “hospitalization”.

***Response: revised to*** *Cardiac events included syncope, palpitations, ventricular arrhythmias, cardiac arrest as a primary diagnosis for hospitalization including death.*

The authors in the introduction state that drugs are a common cause of long QT syndrome. I suggest to specify that they are talking about “acquired long QT syndrome”.

***Response: added acquired as suggested.***

Introduction, line 5: missing space before bracket.

***Response: revised.***

It would be clinically helpful to provide a percentage of adverse events for each risk category (low/moderate/high/very high).

***Response: We do not have the appropriate denominator to calculate the percentage of such events; hence, we included the total number of events by each risk category in Figure 1.***

Heart failure (HF) did not emerge as a predictor of arrhythmia. As authors state, this is a singular finding, as HF is one of the cardiovascular diseases more strictly linked to arrhythmias. However, I would argue in the discussion section, that HF is an extremely heterogeneous condition. Therefore, some phenotype and some aetiology may be more pro-arrhythmic than others. This issue may explain this singular finding.

***Response: Thank you for this feedback. We have added the following sentence in the discussion section****: It is possible that heart failure is a heterogeneous condition and certain phenotypes may be prone to cardiac arrhythmias.*

In the references number 6,11,14, 23, 24, 25 not all authors are listed, even if in some of these the total number of authors does not exced 6. Please format all the references according to the journal policy (list all the authors if the total amount of authors does not excede 6).

***Response: Revised.***

**Reviewer #2**

The study I read seems particularly interesting to me through its conclusions especially in this period when we face the Covid-19 Pandemic, when many SARSCoV2 patients receive Azithromycin alone or in combination with hydroxychloroquine in the hope of amelioration especially of lung damage. Both drugs are known to have a potential for QT prolongation.

First of all, I want to mention that I am not a statistician but a clinical cardiologist. Even so, I consider that the statistical study made by the authors, imagining two statistical models one for the doctor who prescribes azithromycin and the other for the pharmacist who issues the prescription are very correct. And the conclusions that follow are of great help for both the prescribing doctors and the pharmacists.

The studied population is significant in size and includes all age and sex categories but is an outpatient population for which the data is collected from a database belonging to private insurance companies and therefore does not contain clinical parameters such as history of acute myocardial infarction or heart failure or biological markers such as blood ions that would be of interest in this instance. Nevertheless, the authors mention these aspects as limitations of the study. The current study is retrospective and observational using a database conceived for billing purposes and not for research purposes which represents other limit of the study.

However, doctors can use the score derived from the statistical model in identifying high-risk patients based on clinical conditions preceding the prescription of Azithromycin especially syncope and history of palpitations. Pharmacists can also use the score derived from model that takes into account especially previously prescribed drugs that may prolong the QT interval such as antiarrhythmics, antiemetics, antidepressants or loop diuretics.

For these reasons it is of great practical interest to publish this manuscript provided that the imagined statistical models will be developed through a prospective study.

***Response: Thank you for your helpful feedback. No changes were required based on the feedback.***

**Reviewer #3:**

In the paper entitled” Risk of Cardiac Events with Azithromycin – A Prediction Model” by Haridarshan Patel, the authors developed two prediction models, which included baseline demographics, clinical conditions (Model 1), concurrent use of any drug (Model 1) and therapeutic class (Model2) with a risk of QT-prolongation (CQT-Rx) through a splitsample approach, concluded that the Assessment of Cardiac Risk with Azithromycin score maybe helpful to identify patients who are at higher risk of cardiac events following treatment with azithromycin and providers should assess the risk-benefit of using azithromycin and consider alternative antibiotics among high-risk patients.

1. This is an interesting and fairly well-written paper dealing with identifying patients who are at higher risk of cardiac events following treatment with azithromycin through the Assessment of Cardiac Risk with Azithromycin score.

***Response: Thank you. No changes suggested by the reviewer.***

2. It is well known that ACEIs has protective effect on cardiac remodeling. There is a minor issue that needs attention in order to explain the administration of ACEIs predicts a higher risk of cardiac events following administration with Azithromycin.

***Response: Thank you. We have added the following in our discussion to help answer this concern:*** *ACE inhibitors have a protective effect on cardiac remodeling, but the potassium-sparing effects may help explain why it was one of the predictors of cardiac events with azithromycin.*
